# Selective Determination of Glutathione Using a Highly Emissive Fluorescent Probe Based on a Pyrrolidine-Fused Chlorin

**DOI:** 10.3390/molecules28020568

**Published:** 2023-01-05

**Authors:** Francisco G. Moscoso, Carla Queirós, Paula González, Tânia Lopes-Costa, Ana M. G. Silva, Jose M. Pedrosa

**Affiliations:** 1 Departamento de Sistemas Físicos, Químicos y Naturales, Universidad Pablo de Olavide, Ctra. Utrera km. 1, 41013 Seville, Spain; 2 LAQV-REQUIMTE, Departamento de Química e Bioquímica, Faculdade de Ciências da Universidade do Porto, 4169-007 Porto, Portugal

**Keywords:** carboxylated pyrrolidine-fused chlorin, fluorescence, glutathione detection

## Abstract

We report the use of a carboxylated pyrrolidine-fused chlorin (TCPC) as a fluorescent probe for the determination of glutathione (GSH) in 7.4 pH phosphate buffer. TCPC is a very stable, highly emissive molecule that has been easily obtained from meso-tetrakis(4-methoxycarbonylphenyl) porphyrin (TCPP) through a 1,3-dipolar cycloaddition approach. First, we describe the coordination of TCPC with Hg(II) ions and the corresponding spectral changes, mainly characterized by a strong quenching of the chlorin emission band. Then, the TCPC-Hg^2+^ complex exhibits a significant fluorescence turn-on in the presence of low concentrations of the target analyte GSH. The efficacy of the sensing molecule was tested by using different TCPC:Hg^2+^ concentration ratios (1:2, 1:5 and 1:10) that gave rise to sigmoidal response curves in all cases with modulating detection limits, being the lowest 40 nM. The experiments were carried out under physiological conditions and the selectivity of the system was demonstrated against a number of potential interferents, including cysteine. Furthermore, the TCPC macrocycle did not showed a significant fluorescent quenching in the presence of other metal ions.

## 1. Introduction

Chlorins are an important class of reduced porphyrins (dihydroporphyrins) which, when in the form of Mg^2+^ complexes, can be found as the green photosynthetic pigments (chlorophylls) in plants, microalgae and cyanobacteria [1]. As natural chlorins are often unstable and difficult to handle, the preparation of synthetic analogues has been explored as a very attractive approach to obtain more robust derivatives with functional groups suitable for the desired applications [2]. Due to its structural features, chlorin derivatives show unique spectral properties including: (1) an intense absorption band at ca. 650 nm; (2) high emission quantum yield and (3) upon coordination with metal ions, several metallochlorins can be obtained with more valuable physicochemical properties that enable a broader range of applications, such as in theranostics of cancer [3,4,5,6], nonlinear optics [7,8,9], photoacoustic imaging [10,11,12] and for dye sensitised solar cells [13,14,15].

Considering the potential use of chlorin derivatives in biomedical applications, it is important to study their effects in vitro and in vivo systems, including their toxicity, biocompatibility and cellular uptake [16]. Nowadays the great majority of in vitro and in vivo studies are still performed using porphyrin derivatives, nevertheless similar elations can be taken towards chlorin derivatives. For sensing applications in living systems, it is crucial to study and decrease the potential toxicity of the sensors, while maintaining their photophysical properties, like high emission intensity, singlet oxygen (${}^{1}$O${}_{2}$) quantum yields, brightness and luminescence lifetime. In the case of porphyrin and chlorin derivatives, two related factors are extremely important for attaining a sensor with low toxicity, namely the phototoxicity and singlet oxygen generation. A method to decrease the derivatives toxicity is to disable their capacity to penetrate the cellular membranes. For example, in 2011 a dendritic benzoporphyrin was prepared to be used as in vivo oxygen probes and revealed low phototoxicity, this was associated with the bulky structure inability to penetrate the cellular membranes [17]. Another reported method, is the preparation of dyads using dyes that promote the quenching of the triplet state of tetrapyrrolic compounds by energy transfer, disabling the production of singlet oxygen [18]. Another strategy is the preparation of nanoscale structures. Recently, a chlorin-nanoscale-Metal-Organic Framework (TCPCUiO) was reported and revealed applicability both in photodynamic therapy (PDT) and photothermal therapy (PTT). TCPCUiO presented good anticancer activity against H22 tumor-bearing mice in vivo and possessed negligible systematic toxicity—with favorable non thrombogenic and biocompatible properties on blood cells, low systemic toxicities to the function of the liver and kidney and no tissue damage or inflammatory lesions were observed in all major organs [19].

Chlorins can be obtained from porphyrins by a variety of synthetic approches, including through hydrogenation [14], annulation [20] and cycloaddition [21] with diverse entities. Using 1,3-dipolar cycloaddition (1,3-DC) reaction of porphyrins with azomethine ylides [22], we recently reported the synthesis of carboxylated pyrrolidine-fused chlorin (TCPC) and its application in the successful detection of the explosive triacetone triperoxide (TATP) in the gas phase [23].

Glutathione (GSH) is the most abundant thiol in animal cells. It is a tripeptide with a peptide linkage between cysteine (Cys), glycine (Gly) and glutamate (Glu). Cellular GSH exist in the 1–10 mM concentration range whereas it is reduced to 150–200 μM in serum [24] and 1–6 μM in plasma [25,26,27]. The most important role of GSH in the organism is as an antioxidant agent, preventing damage caused by free radicals or reactive oxygen species (ROS). Moreover, GSH is involved in a vast regulatory process, and an abnormal concentration of GSH is directly related to severe diseases [28]. For instance, some tumours have an extraordinarily high level of GSH [29,30,31]. Furthermore, a GSH or GSH synthetase deficiency involves massive urinary excretion, metabolic acidosis, and/or a tendency to hemolysis in humans [28]. It has been also associated with mitocondrial disorders [32], Alzheimer’s and Parkinson’s diseases, among others [33,34,35,36].

For these reasons, it is essential to detect GSH to prevent or downplay the effects of the low or high levels of the tripeptide. For the determination of thiols and specifically GSH, some traditional techniques have been applied, such as high-performance liquid chromatography (HPLC) [37,38,39], surface enhanced Raman scattering (SERS) [39,40] mass spectroscopy (MS) [41] or electrophoresis [42,43]. Although these techniques provide high resolution for low levels of GSH, the cost of equipment, the complexity of sample preparation and extended analysis time means that these analysis methods are not practical for clinical or research purposes.

Electroanalytical methods have also been used to detect GSH [44,45,46]. However, these techniques are based on reducing the thiol group; the detection mechanism is not specific for GSH, and they require sample pre-treatment to isolate the analyte of interest.

**Figure 1:**
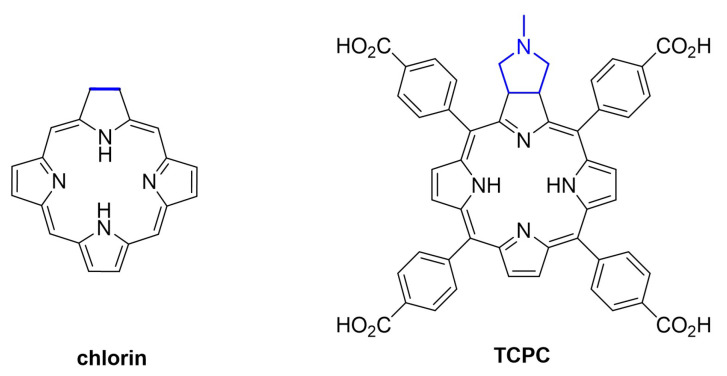
Structure of core chlorin and TCPC.

On the other hand, colorimetric and fluorescent probes have become an excellent alternative for GSH detection due to their simple mechanisms, easy sample preparation, and colour changes with the naked eye when the analyte is present [32,47,48,49,50,51,52,53]. However, taking advantage of these properties requires selective, sensitive and robust methods. In this work, a highly emissive porphyrin analogue, a carboxylated pyrrolidine-fused chlorin (hereafter TCPC, Figure 1), has been synthesised and used as a fluorescent probe for the turn-on-based sensing of GSH fulfilling all the above requirements.

## 2. Results and Discussion

### 2.1. Absorption and Emission Features of TCPC

**Figure 2:**
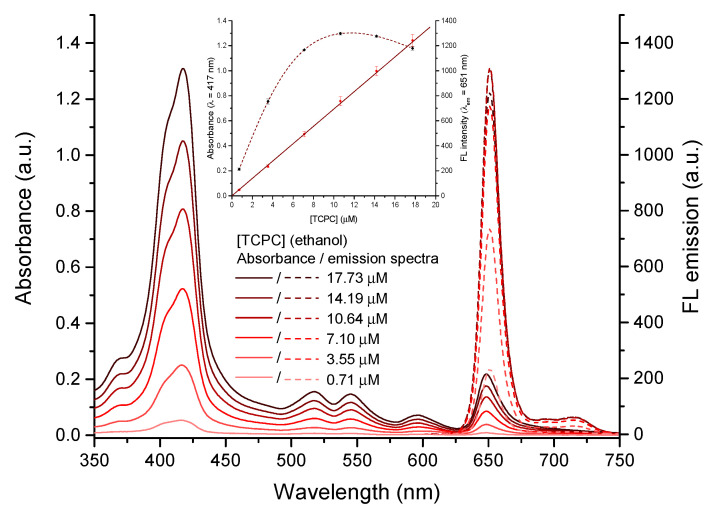
Absorbance (solid lines) and fluorescence ($\lambda_{ex}=415$ nm, dashed lines) spectra of TCPC solutions in ethanol at different concentrations. Inset: Absorbance and FL intensity vs. TCPC concentration. The error bars were calculated from the standard deviation of three independent measurements.

Chlorins are porphyrin derivatives that suffer a single β,β′-double bond reduction, and their spectral features are consequently similar to those of the porphyrin precursor. Here, we used the carboxylated pyrrolidine-fused chlorin (TCPC) as a fluorescent probe, which was prepared as previously reported [23]. Briefly, its synthesis involved the 1,3-dipolar cycloaddition (1,3-DC) of the porphyrin precursor meso-tetrakis(4-methoxycarbonylphenyl)porphyrin TCPP with azomethine ylide, obtained from sarcosine and paraformaldehyde, followed by methyl ester hydrolysis under alkaline conditions. The absorbance and fluorescence spectra of TCPC in ethanolic solution at different concentrations are depicted in Figure 2. The absorbance spectra show the Soret band with a main peak centred at 418 nm and a shoulder at 404 nm. This splitting of the Soret band is attributed to orbital symmetry breaking after alteration of the macrocycle structure that induced a non-degenerate electronic transition [54,55,56,57,58,59,60,61,62]. An enhanced intensity of the Q bands at longer wavelengths is also appreciable [63], in contrast to free-base porphyrins [64]. On the other hand, the emission spectra exhibit the characteristic 0-0 peak at 651 nm and the corresponding 0-1 vibronic band at 715 nm typical of porphyrins. However, the emission intensity of the chlorin shows a six-fold increase as compared to its porphyrinic counterpart TCPP in solutions with the same absorbance (Figures S1 and S2). Regarding the concentration effect, the inset in Figure 2 clearly shows that the linearly increase in the absorbance is accompanied by a decrease in the emission intensity for increasing concentrations of TCPC, which is due to an efficient reabsorption of the photoluminescence at 650 nm where the intense Q band is centered.

### 2.2. TCPC-Hg^2+^ Complex

**Figure 3:**
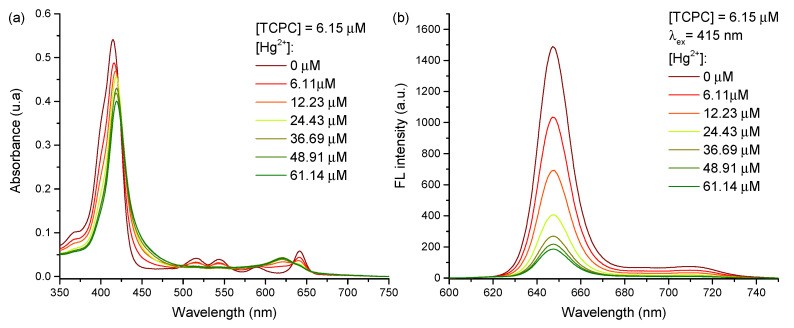
Absorbance (**a**) and fluorescence (λ_*ex*_ = 415 nm) (**b**) spectra of TCPC aqueous solutions at pH 7.4 with different concentrations of Hg^2+^.

Prior to GSH determination experiments, we formed the TCPC-Hg^2+^ complex by adding an increasing amount of HgCl_2_ to the chlorin solution, whose colour gradually turned green as the Hg(II) concentration increased from 0 μM to 61.4 μM (solutions a–g in Figure S3). These colour changes are produced by a red shift of the Soret band and the disappearance of the original Q bands, exhibiting a unique absorption at 620 nm, as shown in Figure 3a. Those spectral changes, typical of protonation of the porphyrin ring, are promoted by the Hg^2+^ coordination to the pyrrolic ring nitrogens, adopting a non-planar conformation [65,66,67]. Moreover, a fluorescence decrease is observed with the formation of the complex, as shown in Figure 3b. The non-planar conformation induced by the mercury ion coordination breaks the π-electrons conjugation of the macrocycle, promoting the excited states of the molecule to relax by non-radiative phenomena, implying a significant quenching of fluorescence emission [68]. The fluorescence quenching follows a linear relationship according to the Stern-Volmer Equation (Figure S4):

(1) $$\frac{I_{0}}{I}=1+K_{SV}\left[Q\right]$$

where $I_{0}$ is the initial FL intensity at 650 nm, *I* is the FL intensity at the same wavelength in the presence of the quencher, $K_{SV}$ is the Stern-Volmer constant and [Q] is the quencher concentration. The results are well-fitted to the Stern-Volmer model indicating that the stoichiometry of the complex remains constant in the working concentration range.

### 2.3. GSH Determination

**Figure 4:**
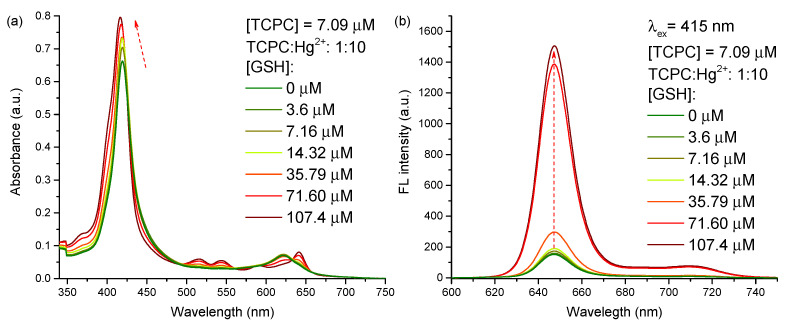
Absorbance (**a**) and fluorescence ($\lambda_{ex}=415$ nm) (**b**) spectra of TCPC-Hg^2+^ complex aqueous solutions (ratio 1:10) at pH 7.4 with different concentrations of GSH.

Due to the excellent luminescent properties of TCPC, it was used as a fluorescent probe for GSH determination. For this approach, three different molar ratios of TCPC-Hg^2+^ were fixed, i.e., 1:2, 1:5 and 1:10. After the TCPC-Hg^2+^ complex formation (logK=5.7 estimated from the absorbance data at 650 nm in Figure 2 and Figure 3, for TCPC:Hg^2+^ = 1:2), increasing amounts of GSH were gradually added to the complex solution. Subsequently, the Hg^2+^ ions are bonded to the thiol groups of two different GSH molecules, forming a [Hg(GSH)_2_]^4−^ complex [69,70,71] (logK=33.4 [72]) (Figure S5). Firstly, the free Hg^2+^ ions coordinate to GSH, causing almost no change in the absorption and fluorescence spectra. Once the mercury ions in excess have been coordinated, the remaining GSH, breaks the TCPC-Hg^2+^ complex due to the higher binding constant of the [Hg(GSH)_2_]^4−^ complex. This reaction liberates the TCPC molecules triggering the fluorescence turn on, as shown in Figure 4 for a TCPC-Hg^2+^ 1:10 molar ratio. In particular, Figure 4a shows that the absorption spectrum is progressively recovered to its initial state as the analyte is added to the sensing solution, confirming the proposed mechanism. At the same time, the corresponding fluorescence spectra (Figure 4b) show an increase of the emission intensity that is used as the response signal. These changes are also visually observed in Figure S6. Once the reaction is completed and the TCPC molecules are quantitatively in their free form, no additional spectral changes are produced for further addition of GSH. A similar behaviour is found when the experiment is performed using the TCPC:Hg^2+^ ratios of 1:2 and 1:5 (Figures S7 and S8, respectively), with the main difference being the minimum amount of GSH needed to obtain the required spectral changes. Therefore, the TCPC-Hg^2+^ ratio determines the minimum amount of analyte to be detected as explained below.

**Figure 5:**
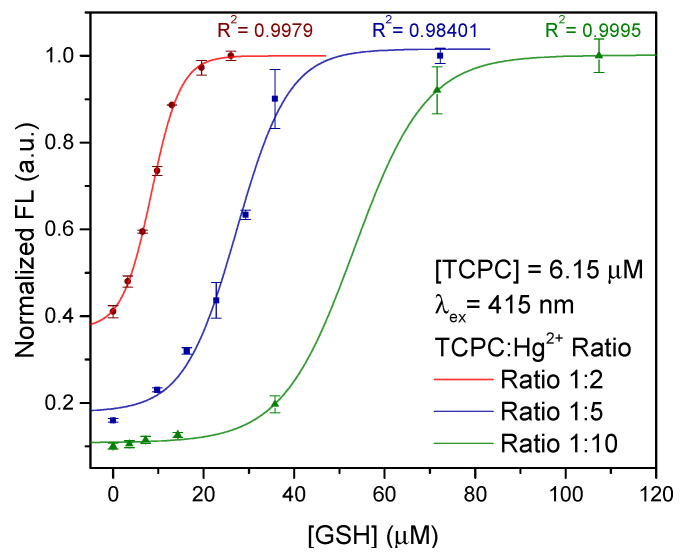
Fluorescence intensity changes when a different amount of GSH is added to (red) 1:2, (blue) 1:5, and (green) 1:10 molar ratio of TCPC-Hg^2+^ complex aqueous solutions at pH 7.4. The error bars were calculated from the standard deviation of three independent measurements.

Figure 5 depicts the normalized fluorescence at the maximum emission wavelength (650 nm, $\lambda_{ex}=415$ nm) of the TCPC molecule vs. the GSH concentration added to the solution for the three-selected TCPC:Hg^2+^ concentration ratios.

As can be seen, the intensity of the signal follows a sigmoidal trend in all cases. Deeper inspection reveals that the position of the curves and the length of the corresponding tails before the intensity takes off are given by the excess amount of Hg^2+^ ions in the media. In this sense, as the TCPC:Hg^2+^ concentration ratio decreases (lower amount of free Hg^2+^ ions in the media) the minimum and maximum concentrations of GSH that can be detected decreases. In all cases, the experimental data can be fitted to the followed equation for calibration purposes:

(2) $$FL=\frac{\alpha }{1+\beta e^{-kc}}+FL_{res}$$

where *c* is the GSH concentration, α, β, and *k* are empirical parameters, and $FL_{res}$ is the residual fluorescence from non-coordinated TCPC in the absence of GSH. As can be seen in Figure 5, excellent values for the regression coefficient were obtained in all cases. Additionally, the corresponding values for ${FL}_{res}$ (0.372, 0.179 and 0.108 for TCPC:Hg^2+^ 1:2, 1:5 and 1:10, respectively) increase as the TCPC:Hg^2+^ ratio decreases. This effect is expected for sufficiently low TCPC:Hg^2+^ ratios due to the presence of non-coordinated insensitive TCPC molecules in the media and reduces the GSH concentration range that can be detected, although it corresponds to low concentration regimes that can be more interesting for certain applications.

**Figure 6:**
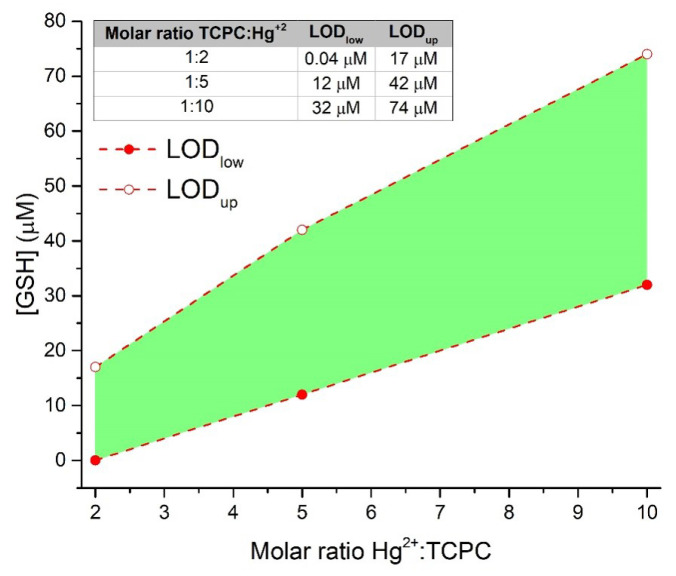
Variation of the LOD${}_{low}$ and LOD${}_{up}$ again the molar ratio of Hg^2+^-TCPC. The green region represents the determination range. Inset: Table of values of LOD${}_{low}$ and LOD${}_{up}$ in μM for the different ratios.

These considerations are better understood if we focus on an essential feature of any analytical method like the limit of detection (LOD). Traditionally, in the context of simple measurements where the signal varies linearly with the amount of analyte, the linear regression method is used and the LOD is defined as 3σ/slope, where σ is the standard deviation of the intercept. However, the complexity of the data that analytical systems can provide for incoming samples leads to situations where the LOD cannot be calculated as reliably as before. In this way, different strategies to calculate the LOD could be considered for optical sensors with a sigmoidal response. In this case, the LOD can be defined as a quarter of the maximum slope of the curve [73], giving rise to two different values of LOD. The first one is the lower quantity of analyte that can be detected, namely the lower limit of detection (LOD${}_{low}$). The second one is the upper limit of detection, LOD${}_{up}$, and represents the maximum amount of analyte that can be detected being the analyte concentration at which the sensor signal is saturated. The LOD${}_{low}$ and LOD${}_{up}$ of the different TCPC:Hg^2+^ molar ratios tested in this study are shown in Figure 6. The green shadowed area represents the [GSH] determination region corresponding to the different concentrations of GSH that the sensor can determine reliably. As can be seen, the TCPC:Hg^2+^ ratio to be employed determines both the LOD${}_{low}$ and LOD${}_{up}$ values and the [GSH] range lying between them. Although these values could be extended to lower and higher GSH concentration regimes by using more extreme TCPC:Hg^2+^ ratios (see Figure S9 for a TCPC:Hg^2+^ 1:100 molar ratio), those used here allow the GSH determination in plasma [24] and serum [25,26,27] samples. Additionally, Figure S9 demonstrates the effective recovery of the TCPC emission after addition of 10 mM GSH. Moreover, applications where sub-micromolar GSH concentrations need to be determined can be covered by this chlorin-based fluorescent probe, with a LOD${}_{low}$ for TCPC:Hg^2+^ 1:2 as low as 40 nM. This value, susceptible to further reduction by reducing the TCPC:Hg^2+^ ratio, is significantly lower than those found in most of the existing literature using fluorescent probes [32,74,75,76,77,78,79,80,81] and very similar to those reporting the lowest values [53,67,75]. These results are summarized in Table S1.

**Figure 7:**
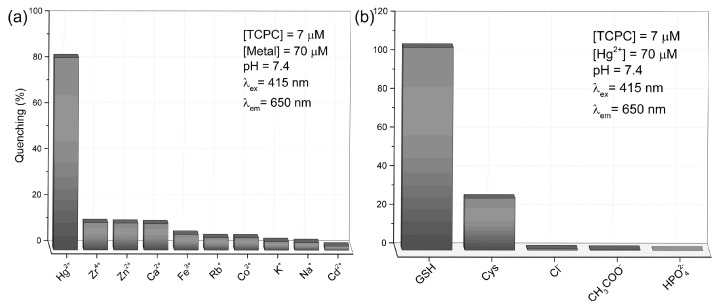
(**a**) Fluorescence quenching of TCPC in the presence of different metal ions. The experimental conditions are shown in the figure. (**b**) Recovery of the TCPC fluorescence when GSH, cys and other ions were added. The experimental conditions are shown in the figure. GSH and cys are in physiological concentration. Other ions are in 1 equivalent to the GSH.

Finally, we carried out the selectivity study selecting two families of potential interferents: metal ions that could compete with Hg^2+^ to quench the chlorin emission, and competing thiols and other common anions (ligands) present in biological samples. Figure 7a shows the response of TCPC against ten equivalents of different metal ions. As can be seen, only Hg^2+^ is able to quench the fluorescence of the TCPC by the mechanism discussed above, even with ten-fold concentrations of the potential interferents. Those results reveal the high selectivity of the TCPC towards mercury ions. On the other hand, Figure 7b shows the results for cysteine (cys), Cl^−^, acetate and phosphate dibasic (HPO_4_^2−^). In this experiment, GSH is ten times more concentrated than cys in order to simulate physiological concentrations, while the other ions are in 1 equivalent to the GSH. As can be seen, only cys can produce an important interference in the proposed mechanism. In fact, cys is the most plausible interferent in an intracellular or extracellular medium. As discussed above, this amino acid is part of the structure of glutathione, and the thiol group could coordinate with mercury ions in the media, giving rise to a false positive. However, in the human organism, the concentration of cys is between 30 and 50 times lower than that of GSH [25,82] and therefore, it does not represent real interferent in detecting GSH.

## 3. Materials and Methods

### 3.1. Chlorin, Reagents and Instrumentals

The synthetic procedure and characterization (including ${}^{1}$H NMR, ${}^{13}$C NMR, and HRMS) for TCPC is described elsewhere [23]. Glutathione and mercury(II) chloride (HgCl_2_ > 99.5%) were purchased from Sigma-Aldrich. Other reagents and solvents were purchased as reagent-grade and used without further purification. UV-visible absorbance spectra were recorded using a Cary 100 UV-Vis spectrophotometer (Agilent Technologies, Santa Clara, CA, USA). In addition, fluorescence (FL) emission and excitation spectra were recorded with a Hitachi F-7000 Fluorescence Spectrophotometer (Hitachi, Tokyo, Japan).

### 3.2. Sensing Experiments

GSH fluorescence determination was carried out by increasing the GSH concentration in a TCPC-Hg^2+^ complex aqueous solution to obtain a calibration curve. To ensure the maximum formation of TCPC-Hg^2+^, the experiments were performed with different TCPC-Hg^2+^ molar ratios (1:2, 1:5 and 1:10). All experiments were carried out by fixing the pH to 7.4 using a phosphate buffer (H_2_PO^4−^/HPO_4_^2−^) 0.01 M. All determination assays were carried out at $\lambda_{ex}=415$ nm, close to the absorption peak of TCPC.

## 4. Conclusions

The chlorin TCPC exhibits appealing emissive features that prompted us to use it in chemical sensing. In particular, we employed the TCPC molecule as a fluorescent probe to detect different concentrations of GSH with high reliability and sensitivity, being able to detect even 40 nM of GSH in physiological conditions. For this approach, a controlled amount of HgCl_2_ is added to the solution to form the non-emissive Hg^2+^ complex which, in turn, is broken in the presence of GSH by the formation of the [Hg(GSH)_2_]^4−^ complex, releasing free TCPC molecules and triggering the fluorescence turn on. This increase in the fluorescence intensity accurately fits a sigmoidal curve as a function of the GSH concentration, which enables the proposed method to perform quantitative analysis. Furthermore, the specificity of TCPC for the Hg^2+^ coordination and the selectivity of the TCPC-Hg^2+^ complex against GSH was demonstrated.

## Data Availability

Not applicable.

## Supplementary Materials

The following supporting information can be downloaded at: https://www.mdpi.com/article/10.3390/molecules28020568/s1, Figure S1: Absorption spectra of a methanol solution of meso-tetrakis(4-carboxyphenyl)porphyrin (TCPP) at different concentrations. Inset: linear fitting of the maximum absorbance vs. [TCPP].; Figure S2: Fluorescence emission spectra ($\lambda_{ex}=415$ nm) of a methanol solution of meso-tetrakis(4-carboxyphenyl)porphyrin (TCPP) at different concentrations. Inset: FL emission intensity vs. [TCPP].; Figure S3: TCPC ethanolic solutions with different concentrations of Hg^2+^ (from A to G: 0, 6.11, 12.23, 24.43, 36.69, 48.91, 61.14 μM). See the main text for further details.; Figure S4: TCPC ([TCPC] = 6.15 μM) fluorescence dependence with Hg^2+^ concentration (black dots) and its Stern-Volmer equation linear fitting (red line), $\lambda_{ex}=415$ nm, $\lambda_{em}=650$ nm; Figure S5: Schematic representation of the complex [Hg(GSH)_2_]^4−^ (see [69] in main article).; Figure S6: Photographs of cuvettes containing a TCPC aqueous solution (left), its corresponding TCPC−Hg^2+^ complex (center) and after addition of an excess of GSH (right) under visible (a) and UV (b) light.; Figure S7: Absorbance (left) and fluorescence ($\lambda_{ex}=415$ nm) (right) spectra of TCPC−Hg^2+^ complex aqueous solutions (ratio 1:2) at pH 7.4 with different concentrations of GSH.; Figure S8: Absorbance (left) and fluorescence ($\lambda_{ex}=415$ nm) (right) spectra of TCPC−Hg^2+^ complex aqueous solutions (ratio 1:5) at pH 7.4 with different concentrations of GSH.; Table S1: Limit of detection and response rates of various fluorescent sensors toward GSH.
