# Supplementary material for: Selective Determination of Glutathione Using a Highly Emissive Fluorescent Probe Based on a Pyrrolidine-Fused Chlorin
Source: Molecules. 2023 Jan 5;28(2):568. doi: 10.3390/molecules28020568 (PMC9862258; doi:10.3390/molecules28020568)
Supplement: Supplementary file 1 [file molecules-28-00568-s001.zip › molecules-2082428-supplementary.pdf]

Supporting Information for

Selective Determination of Glutathione Using a Highly Emissive Fluorescent Probe Based on a Pyrrolidine-Fused Chlorin

Francisco G. Moscoso [1,*] , Carla Queirós [2] , Paula González [1], Tânia Lopes-Costa [1] , Ana M. G. Silva [2] and Jose M. Pedrosa [1,*]

[1] Departamento de Sistemas Físicos, Químicos y Naturales, Universidad Pablo de Olavide, Ctra. Utrera km. 1, 41013 Seville, Spain

[2] LAQV-REQUIMTE, Departamento de Química e Bioquímica, Faculdade de Ciências da Universidade do Porto 4169-007 Porto, Portugal

[*]Corresponding authors: fjgarmos@upo.es jmpedpoy@upo.es

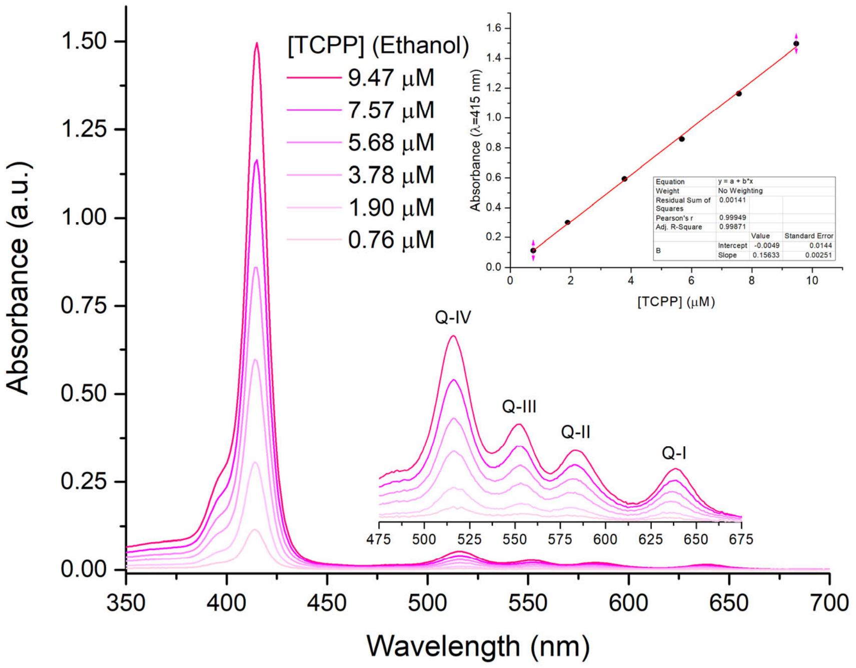

Figure S1. Absorption spectra of a methanol solution of *meso*-tetrakis(4-carboxyphenyl)porphyrin (TCPP) at different concentrations. Inset: linear fitting of the maximum absorbance vs. [TCPP].

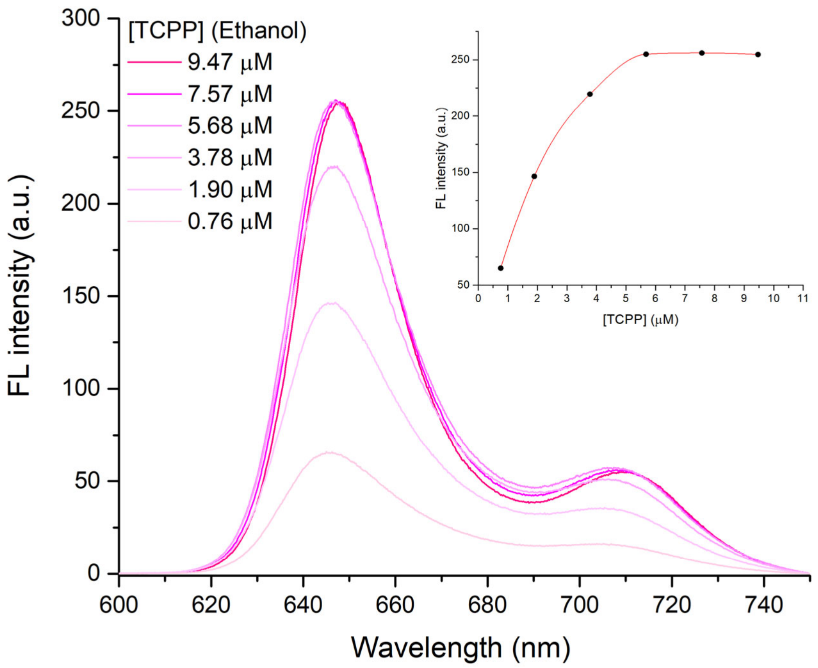

Figure S2. Fluorescence emission spectra (λ_{ex}= 415 nm) of a methanol solution of *meso*-tetrakis(4-carboxyphenyl)porphyrin (TCPP) at different concentrations. Inset: FL emission intensity *vs.* [TCPP].

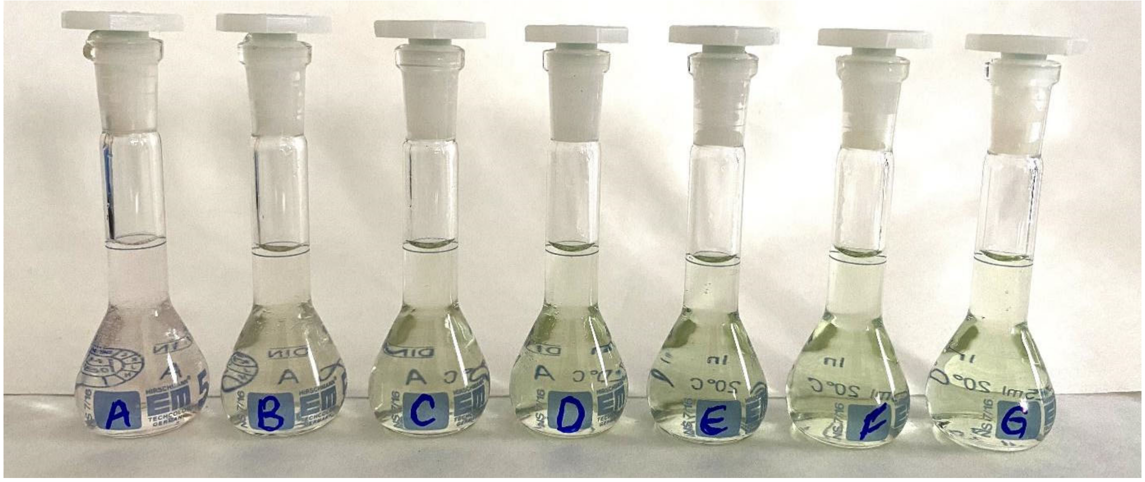

Figure S3. TCPC ethanolic solutions with different concentrations of Hg^{2+} (from A to G: 0, 6.11, 12.23, 24.43, 36.69, 48.91, 61.14 μM). See the main text for further details.

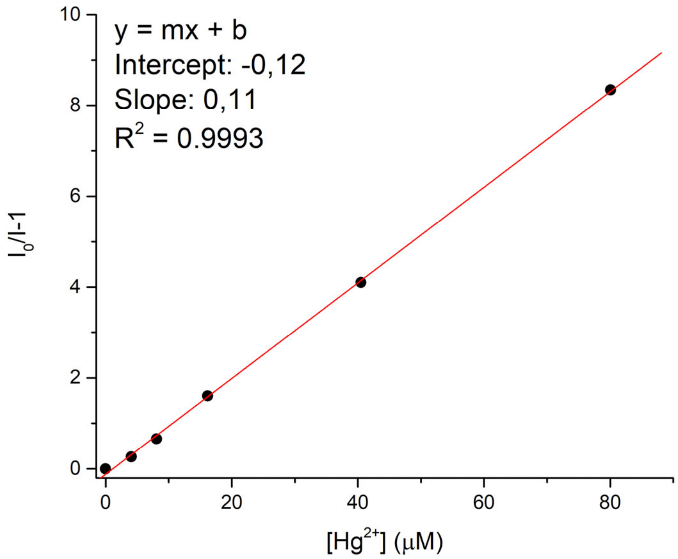

Figure S4. TCPC ([TCPC]=6.15 μM) fluorescence dependence with Hg^{2+} concentration (black dots) and its Stern-Volmer equation linear fitting (red line), λ_{ex} = 415 nm, λ_{em} = 650 nm.

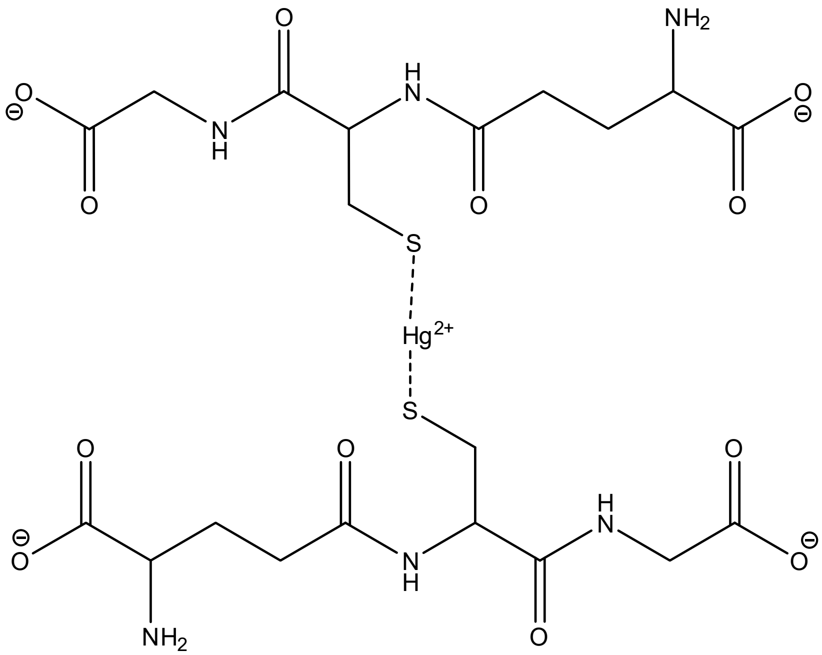

Figure S5. Schematic representation of the complex $[Hg(GSH)_2]^{4-}$ (see reference 69 in main article).

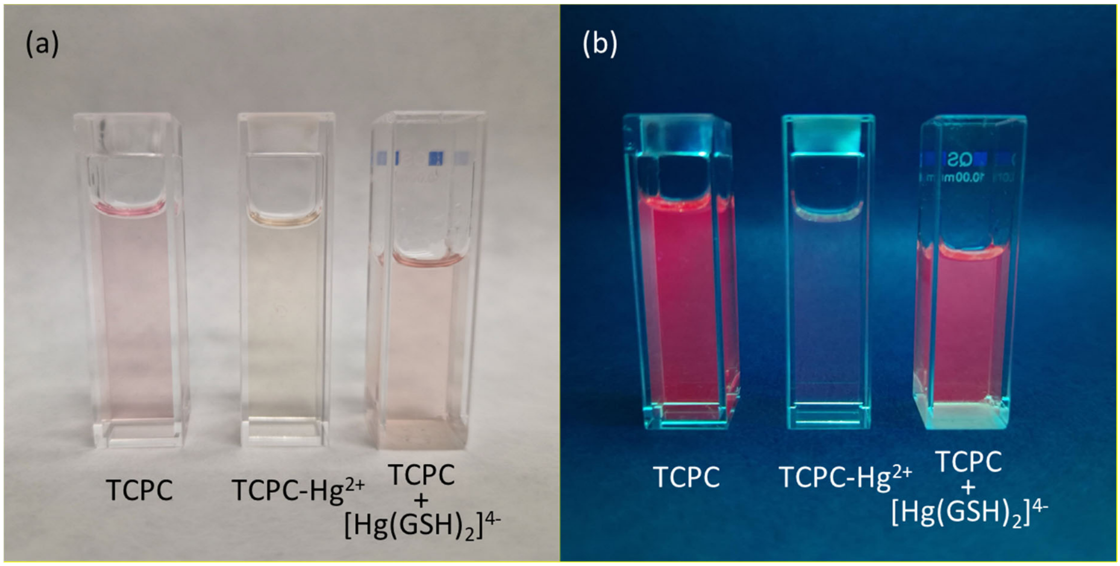

Figure S6: Photographs of cuvettes containing a TCPC aqueous solution (left), its corresponding TPCP-Hg^{2+} complex (center) and after addition of an excess of GSH (right) under visible (a) and UV (b) light.

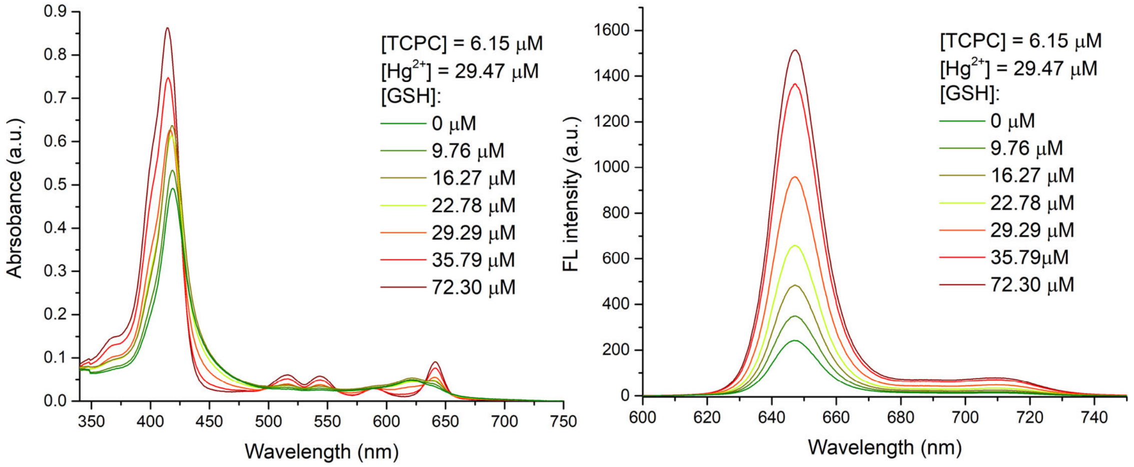

Figure S7. Absorbance (left) and fluorescence (λex = 415 nm) (right) spectra of TCPC-Hg^{2+} complex aqueous solutions (ratio 1:2) at pH 7.4 with different concentrations of GSH.

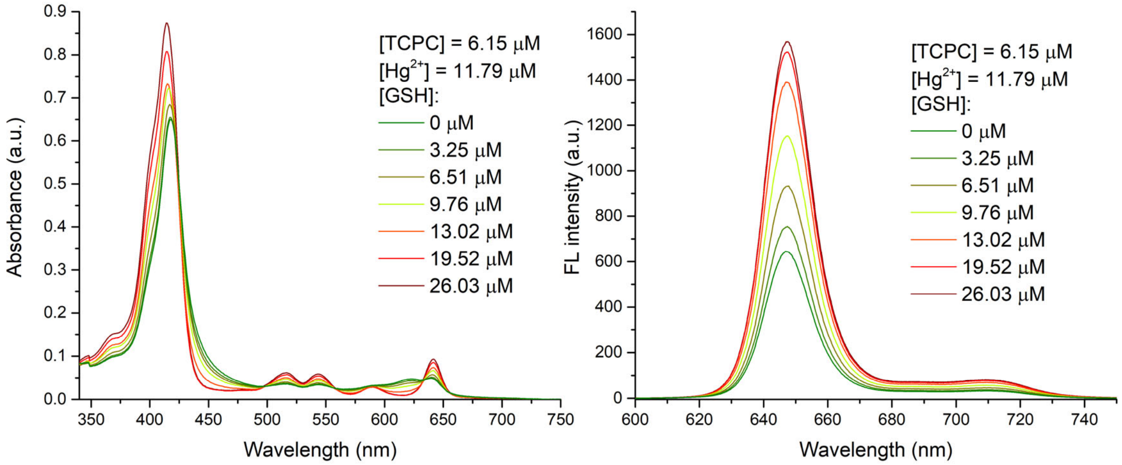

Figure S8. Absorbance (left) and fluorescence (λex = 415 nm) (right) spectra of TCPC-Hg^{2+} complex aqueous solutions (ratio 1:5) at pH 7.4 with different concentrations of GSH.

Table S1. Limit of detection and response rates of various fluorescent sensors toward GSH.

Reference	LOD	Response rate
This study	40 nM	Seconds-order
32	1.35 mM	3-4 minutes
78	1.2 μM	Not reported
80	0.3 μM	Not reported
81	0.89 μM	Not reported
82	2.2 μM	Not reported
83	0.48 μM	Not reported
84	0.96 μM	Not reported
53	16.2 nM	Not reported

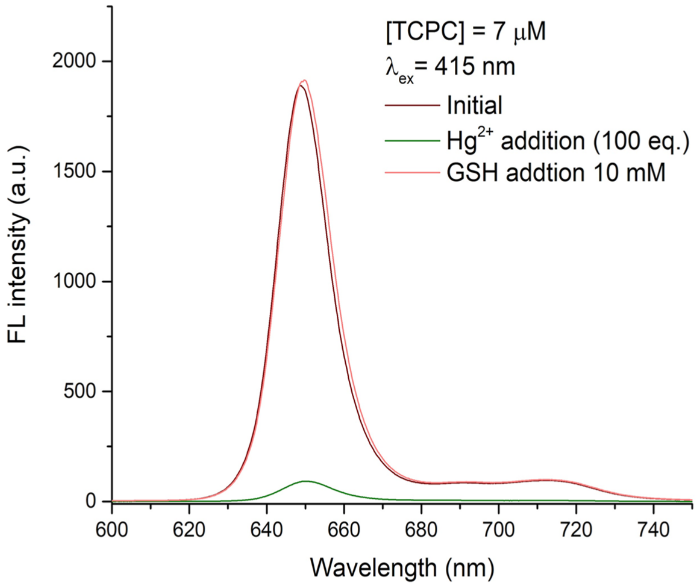

Figure S9. Fluorescence (λex = 415 nm) spectra of aqueous solutions of TCPC, and the TCPC-Hg^{2+} complex (ratio 1:100) before and after addition of 10 mM GSH at pH 7.4.
